# Resveratrol Modulation of Apoptosis and Cell Cycle Response to Cisplatin in Head and Neck Cancer Cell Lines

**DOI:** 10.3390/ijms22126322

**Published:** 2021-06-12

**Authors:** Marinela Bostan, Mirela Mihaila, Georgiana Gabriela Petrica-Matei, Nicoleta Radu, Razvan Hainarosie, Cristian Dragos Stefanescu, Viviana Roman, Carmen Cristina Diaconu

**Affiliations:** 1 Center of Immunology, Stefan S. Nicolau Institute of Virology, 030304 Bucharest, Romania; marinela.bostan@virology.ro (M.B.); mirela.mihaila@virology.ro (M.M.); 2 Department of Immunology, Victor Babes National Institute of Pathology, 050096 Bucharest, Romania; 3 Personal Genetics-Medical Genetics Center, Department of Cytogenetics, 010987 Bucharest, Romania; gabryela.matei@gmail.com; 4 Department of Biotechnology, University of Agronomic Sciences and Veterinary Medicine of Bucharest, 011464 Bucharest, Romania; 5 Biotechnology Department, National Institute for Chemistry and Petrochemistry R&D of Bucharest, 060021 Bucharest, Romania; 6 Otorhinolaryngology and Head and Neck Surgery Department—Carol Davila University of Medicine and Pharmacy, 020021 Bucharest, Romania; razvan@riaclinic.com (R.H.); dragos.stefanescu@umfcd.ro (C.D.S.); 7 Department of Cellular and Molecular Pathology, Stefan S. Nicolau Institute of Virology, 030304 Bucharest, Romania; directie@virology.ro

**Keywords:** resveratrol, cisplatin, head and neck carcinoma line, *c-MYC*, *BCL-2*, *TP53*, *MDM-2*, cell cycle, apoptosis

## Abstract

In head and neck cancers, the effectiveness of cisplatin (CisPt) treatment is limited by its toxicity, especially when higher doses are necessary, and the possible occurrence of cisplatin resistance. This study evaluated the effects of resveratrol (RSV) on the expression of different genes involved in the response of human tumor cells (FaDu, PE/CA-PJ49) to cisplatin therapy. Our results revealed that RSV induced apoptosis amplification in both FaDu and PE/CA-PJ49 cells and modulated the expression of specific genes differently than in normal HaCaT cells. In FaDu cells, combined CisPt + RSV treatment induced an increase in apoptosis, which was associated with an increase in *c-MYC* and *TP53* and a decrease in *BCL-2* expression. While CisPt + RSV treatment induced apoptosis in PE/CA-PJ49 cells by inhibition of *BCL-2* associated with high levels of *MDM-2* and subsequently led to inhibition of *TP53* gene expression. Decreased *c-MYC* expression in PE/CA-PJ49 treated with CisPt + RSV was accompanied by cell cycle blockage in G0/G1 phase. In conclusion, RSV influences tumor cell response to CisPt by inducing apoptosis and modulating gene expression. In addition, in normal HaCaT cells, RSV was able to reduce the harmful effects of CisPt.

## 1. Introduction

On a global level, cancer was and remains to this day a serious matter of public health. It is a complex, multifactorial disease and, despite the evolution of radiotherapy and chemotherapy, there still is no significant progress regarding its treatment. Human head and neck squamous cell carcinoma (HNSCC) is an aggressive type of cancer that develops from epithelial cells. HNSCC is characterized by rapid progression and metastases associated with a poor prognosis [1]. For this type of cancer, chemotherapy is a method to reduce the tumor’s mass before surgery and to prevent metastases afterward. For patients diagnosed in advanced stages of HNSCC that are declared inoperable, chemotherapy remains one of the few therapeutic options. Thus, how tumoral cells respond to chemotherapeutic agents and the methods through which chemotherapy sensibility can be increased represent a major interest both for research as well as for clinical approach [2,3,4]. Conventional therapies for HNSCC consist of treatment with CisPt, which is frequently accompanied by severe adverse reactions, such as nephrotoxicity, ototoxicity, hematologic toxicity, or central nervous system afflictions, that eventually will lead to stopping the treatment [5,6,7]. CisPt diffuses passively through the cellular membrane and is activated in the cellular environment, becoming a strong electrophile, capable of reacting with some of the nucleophilic sites found in the nucleic acids. These reactions between the platinum atom of CisPt and the purine bases found within the DNA lead to the formation of intra-strand and inter-strand covalent bonds [8,9]. These processes allow the formation of CisPt-DNA adducts, which interfere with DNA replication and transcription [10]. Additionally, the interactions between CisPt and some proteins considered to be sulfur donors (such as cysteine, methionine, thiols, etc.) have been described, therefore allowing an understanding of how CisPt enters the cell and interacts with different cellular structures, thus leading to a better understanding of the action mechanism of CisPt and dose efficiency [11,12].

Moreover, this information opens the path for new approaches regarding combined therapies that use different adjuvants with either a protective role or a synergistic antitumoral role. When CisPt induces an advanced degree of deterioration of the DNA, the reparatory capacity of the cells is exceeded and then the cell, through the activation of some specific signaling pathways, enters the stage of programmed cell death, also known as apoptosis [13,14]. Understanding the cytotoxic mechanisms induced by CisPt allows a more precise approach to antitumoral therapies and better management of their efficiency by using natural compounds that can enhance the effects of CisPt, while also reducing its toxicity within the normal cells. In order to reduce the number of chemotherapeutic drug adverse reactions and their magnitude, some natural compounds have been used as adjuvants for chemotherapeutic agents in different in vitro studies and also in many clinical trials [15,16,17,18].

Natural compounds have been used to obtain new drugs, or they have been used for the improvement of the therapeutic scheme in different illnesses in combination with chemotherapeutic drugs. One of the most studied natural compounds is RSV, due to its antioxidant, antitumoral, and anti-inflammatory properties [19,20,21,22]. RSV has drawn significant attention from scientists, as it is capable of suppressing the proliferation of tumoral cells, inducing apoptosis, or of sensitizing tumoral cells to the effects of radio- and chemotherapy [23,24,25,26,27,28]. RSV is a polyphenolic phytoalexin that is found in red fruits, peanuts, and grapes and presents antimicrobial, antioxidant, cardioprotective, and antitumoral properties, a fact that is supported by various in vivo and in vitro studies conducted on a variety of types of cancer such as colon, pancreatic, breast, liver, prostate, or lung cancer [29,30,31,32]. Numerous studies have shown that RSV has a role in modulating the intracellular molecular processes involved in stopping the cell cycle or in apoptosis in different types of cancer, including HNSCC [33,34,35,36,37].

Some genes have an important role in the appearance and evolution of tumoral cells, genes that are involved in signal transduction pathways and thus can control the cellular processes, such as proliferation, cell cycle, and apoptosis induction [38,39,40,41]. Apoptosis, also known as programmed cell death, is a process used to eliminate deteriorated or abnormal cells through alterations of the cellular membrane, cellular contraction, nuclear DNA fragmentation, and apoptotic bodies. Disruption in the regulation of apoptosis is crucial in the development of the tumor and genes such as *TP53*, *MDM-2*, *Bcl-2*, and *C-MYC* play a major role in the process of controlling apoptosis [42,43,44,45,46,47].

A key mediator in preventing carcinogenesis is *TP53*, a tumoral suppressor gene strongly connected to the processes of transcription, proliferation, and apoptosis [48,49]. As an answer to DNA deterioration, *MDM-2*—a ubiquitin ligase E3—can reduce cellular proliferation and can activate apoptosis by targeting *TP53. MDM-2* and *TP53* are both important players involved in DNA damage repair [50,51,52,53]. Another important role in cellular proliferation, cell cycle, apoptotic process, and also in the response to therapy is apparently held by *c-MYC*, a nuclear transcription factor. Data published in different studies regarding the functions of *c-MYC* in different malignant tumors are extremely controversial [54,55,56,57,58,59]. *C-MYC* seems to be involved in the activation of cellular proliferation, as well as in triggering the apoptotic process, without researchers knowing exactly what is determining this dual behavior. Some studies have shown that the B-cell lymphoma gene (*BCL-2*) is overexpressed and has a role in the development of cytostatic resistance in tumoral cells [60,61,62,63].

The *c-MYC* gene could cooperate with other oncogenes such as *BCL-2*, and thus it might influence the mechanisms involved in tumor surveillance. Therefore, targeted therapy against *BCL-2* can enhance the antitumoral effects of chemotherapy by tumoral cell sensitization in order to cause them to enter apoptosis. This study analyzed how RSV treatment alone or in combination with CisPt influences the expression of these genes, as well as the manner in which cellular processes such as the cell cycle, proliferation, and apoptosis of tumoral cells can be modulated. To acquire useful information that could lead to a more efficient therapeutic approach in head and neck cancers, we analyzed the effect of RSV treatment on the response induced by CisPt in FaDu and PE-CA/PJ49 tumoral cell lines. The HaCaT cell line was used in order to analyze the effect of the treatment with RSV and/or CisPt on a normal cell line. The cells were treated both separately and in combination with RSV and/or CisPt, and the effect on the expression of the genes (*c-MYC, BCL-2, MDM-2,* and *TP53*) with a role in the proliferation process, the evolution of the cell cycle, and the activation of the apoptotic process was analyzed. Emphasizing the role of RSV in the modulation of the effect induced by CisPt on the expression of the above mentioned genes could lead to a better understanding of how a cell engages in either the proliferation process or in apoptosis. All the previously mentioned aspects may contribute to the opening of more efficient therapeutic approaches for head and neck cancer.

## 2. Results

### 2.1. RSV Effects on the Proliferation of Head and Neck Tumor Cells Treated with CisPt

We compared the inhibitory effects of RSV and/or CisPt on the proliferation of PE/CA-PJ49, FaDu tumor adherent cells versus HaCaT—normal adherent cells—using the 3-(4,5-dimethylthiazol-2-yl)-2,5- diphenyltetrazolium bromide (MTT) assay. To select the optimal working concentrations, the tumor and normal cells were treated for 24, 48, and 72 h with RSV or CisPt in concentrations ranging between 2 and 256 µM. The drug response curve was generated, and IC50 (the concentration required to kill 50% of the cell population) was calculated using GraphPad Prism version 7.00 [64,65]. Data obtained when treating the cells with RSV or Cis for 48 or 72 h did not differ significantly compared to the values obtained at 24 h (Table 1).

To establish the degree of selectivity of the CisPt or RSV after 24 h treatment we used the IC50 value in order to calculate the selectivity index (SI). SI indicates the cytotoxic selectivity for CisPt or RSV against cancer cells versus normal cells (Table 1) [66,67]. To minimize the toxic effect of CisPt on normal cells and keep them in accord with selectivity index values we selected the working concentration of 10 µM CisPt and 50 µM RSV and the optimal treatment time of 24 h.

As seen in Table 1, the response of tumor cells to CisPt or RSV treatment was different and dependent on agent concentration compared to the response of normal cells. The SI values obtained showed that PJ49 PE/CA tumor cells (SI = 2.63) had a higher selectivity for CisPt treatment than FaDu tumor cells (SI = 1.97). In the case of RSV treatment, both tumor lines had high selectivity, the registered SI being higher than 2.

To determine how the 10 μM CisPt or 50 μM RSV treatment alone or in combination for 24 h influenced the proliferation process of the tumor and normal cells we analyzed the growth rate of the cells using the proliferation kit described in Section 4, Materials and Methods.

The index of proliferation (IP) data obtained when treating the cells for 48 or 72 h did not differ significantly compared to the IP values obtained at 24 h. For this reason, all experiments in our study were performed using concentrations of 50 µM RSV and/or 10 µM CisPt for 24 h.

**Figure 1:**
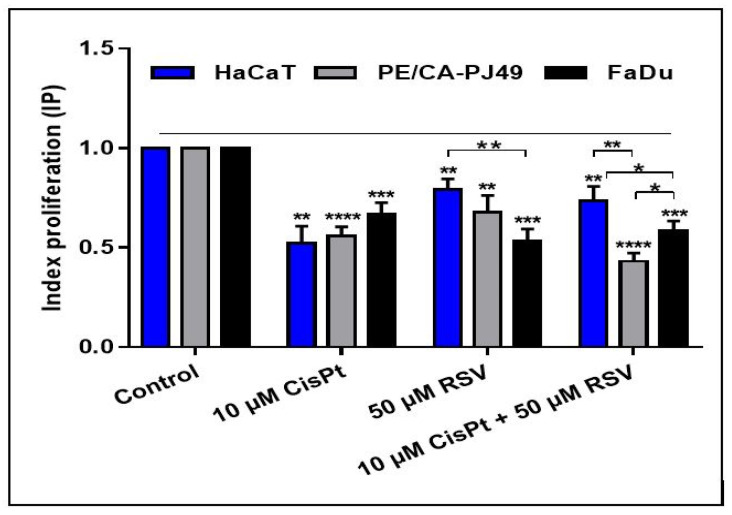
The index of proliferation (IP) for tumor cell line PE/CA-PJ49, FaDu and normal cell line HaCaT treated with RSV and/or CisPt for 24 h. IP is equal to absorbance of treated cells/absorbance of untreated cells. Results are expressed as absorbance mean values of three determinations +/− standard deviation (SD). Untreated cells are considered to have an IP equal 1. (* *p* < 0.05, ** *p* < 0.005, *** *p* < 0.0005; **** *p* < 0.00005).

As seen in Table 2 and Figure 1, treatment with 10 µM CisPt for 24 h reduced the proliferation of PE/CA-PJ49 (IP = 0.55; *p* < 0.00008, ****), FaDu tumor cells (IP = 0.71; *p* < 0.00051, ***) and greatly affected the proliferative process in normal HaCaT cells (IP = 0.67; *p* < 0.00063, ***) compared to untreated cells (Control, IP = 1). The results showed that PE/CA-PJ49 tumor cells respond differently to 10 µM CisPt treatment when compared to FaDu tumor cells (*p* < 0.05; *). A treatment of 50 µM RSV for 24 h affected the proliferation of FaDu tumor cells (IP = 0.54; *p* < 0.00018, ***) more than it did the PE/CA-PJ49 tumor cells (IP = 0.65, *p* < 0.00275, **) and had less influence on the proliferative process of normal HaCaT cells (IP = 0.83, *p* < 0.0022, **) compared to untreated cells (control, IP = 1) (Table 2 and Figure 1).

When the 10 µM CisPt + 50 µM RSV combined treatment was applied, a decrease of proliferation was observed in PE/CA-PJ49 tumor cells (IP = 0.43), the dominant effect on proliferation being exerted by CisPt (*p* < 0.011; *). In contrast, in the case of FaDu tumor cells (IP = 0.59) CisPt + RSV combined treatment reduced proliferation in the same manner as RSV (IP = 0.54; *p* = ns) or CisPt (IP = 0.71; *p* = ns) alone (Figure 1, Table 2). In normal HaCaT cells, data obtained when the combined treatment was applied showed that RSV (IP = 0.83; *p* < 0.029; **) appeared to protect cells from the toxic effect of CisPt (0.67) (Figure 1 and Table 2). As seen in Figure 1, the proliferation of PE/CA-PJ49 tumor cells compared to FaDu tumor cells was affected differently in the case of treatment only with CisPt (*p* < 0.025; *) or RSV (*p* < 0.044; *) applied separately. Comparative analysis of the proliferative response of PE/CA-PJ49 (*p* < 0.00038, ***) or FaDu (*p* < 0.004; **) tumor cells versus the normal HaCaT cells to the combined CisPt + RSV treatment showed a significantly different cellular behavior. It can also be seen that the response of the two tumor cell lines, PE/CA-PJ49 and FaDu, to the CisPt + RSV combined treatment, was significantly different (*p* < 0.05; *).

### 2.2. The RSV Effects on the Gene Expression of Head and Neck Tumor Cells Treated with CisPt

#### 2.2.1. *TP53* Gene Expression Analysis

The action mechanism of cisplatin is not yet fully understood, its effects seem to be partially associated with the *TP53* suppressor gene, which has an important role in cancers. In numerous types of cancer, head and neck cancer included, *TP53* suffers mutations that could have a major impact on the pathology of the disease and its response to treatment. Data from literature support the fact that when *TP53* suffers mutations, the illness becomes aggressive and resistant to ionizing radiation and even to chemotherapy, but there are also opposing opinions regarding the influence of *TP53* mutations on the effectiveness of therapy with cisplatin [68,69].

In order to obtain useful information on the most efficient therapeutic approach in head and neck cancers, we analyzed the effect of treatment with RSV on the response induced by CisPt treatment on FaDu tumor cells that have a point mutation of *TP53* [70] compared to PE/CA-PJ49 tumor cells that have no mutations of the *TP53* gene. The HaCaT cellular line was used to analyze the effect of treatment with RSV and/or CisPt on normal cells.

**Figure 2:**
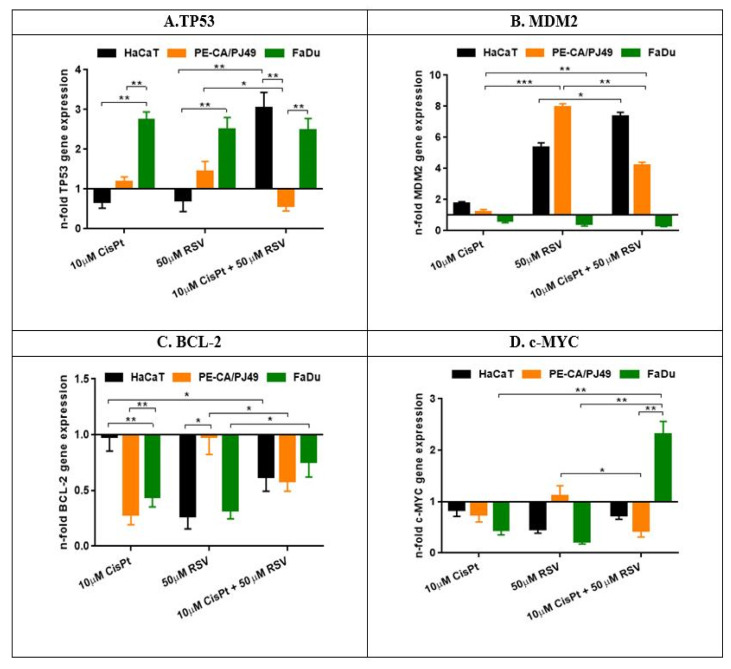
The effect of CisPt and/or RSV treatment on gene expression ((**A**)—TP53; (**B**)—MDM-2; (**C**)—BCL-2; (**D**)—C-MYC) in tumor cells PE/CA-PJ49 and FaDu compared to normal HaCaT cells. The results were obtained using gene reference HPRT (hypoxanthine phosphoribosyltransferase). Control is represented by the untreated cells of each cell line and the n-fold expression for each gene analyzed in control cells is 1. Each sample was performed in duplicate. The samples were analyzed using the formula 2-ΔΔCt = gene expression. (* *p* < 0.05, ** *p* < 0.005, *** *p* < 0.0005).

As shown in Figure 2A, treatment with CisPt 10 µM did not significantly modify the expression of the *TP53* gene in PE/CA-PJ49 tumor cells (*p* = ns). In FaDu tumor cells, treatment with 10 µM CisPt induced a significant growth of *TP53* gene expression (*p* < 0.006, **), compared to the untreated FaDu cells. The cellular response to CisPt treatment showed a significant increase in *TP53* expression in FaDu cells (*p* < 0.007, **) compared to PE/CA-PJ49 cells. In normal HaCaT cells, treatment with 10 µM CisPt led to a decrease of the *TP53* gene expression, compared to the FaDu tumor cells (*p* < 0.005, **) (Figure 2A, Table 3).

The expression of the *TP53* gene was not significantly affected by treatment with 50 µM RSV in the normal HaCaT cells or PE/CA-PJ49 tumor cells. On the other hand, RSV induced a highly significant rise of *TP53* gene expression in FaDu cells, compared to normal HaCaT cells (*p* < 0.009, **) and PE/CA-PJ49 tumor cells (*p* < 0.05, *) (Figure 2A, Table 3).

In the case of the combined treatment RSV + CisPt the expression of the *TP53* gene remained increased in FaDu cells, but without recording an additive effect of the two agents (Figure 2A). The combined treatment with RSV + CisPt on PE/CA-PJ49 tumor cells displayed a significant decrease of the *TP53* expression, compared to FaDu tumor cells (*p* < 0.007, **) and to HaCaT cells (*p* < 0.004, **). The statistical analysis on the effect induced by simultaneous treatment with CisPt + RSV on PE/CA-PJ49 tumor cells, compared to the effects induced by CisPt (*p* < 0.05, *) or RSV (*p* < 0.04, *) separately, showed inhibition of *TP53* expression (Figure 2A, Table 3).

Also, it was observed that combined treatment with CisPt + RSV enhanced the expression of *TP53* in normal HaCaT cells, even though separately they both inhibited *TP53* expression. The ANOVA analysis sustained the different ways in which the combined treatment with CisPt + RSV (*p* < 0.006, **) modified the *TP53* gene expression, compared to the effect induced separately by CisPt or RSV in HaCaT cells (Figure 2A, Table 3).

#### 2.2.2. *MDM-2* Gene Expression Analysis

The link between *TP53* and *MDM-2* is one of the most studied connections between a tumor suppressor gene and an oncogene, both being predisposed to mutations in head and neck cancers. Because the *MDM-2* gene is known as a negative regulator of the *TP53* gene, efforts have been made to establish a direct connection between treatment with CisPt and/or RSV and the level of expression of the two genes. Our study analyzed the effect induced by treatment with CisPt and/or RSV on the expression of the *MDM-2* gene in tumor cells, and we tried to establish a possible link between the *TP53* gene expression and the *MDM-2* gene expression. The obtained results showed that treatment of FaDu cells with 10 µM CisPt and/or 50 µM RSV induced a slight inhibition of *MDM-2* gene expression, compared to untreated cells (Figure 2B, Table 3).

The results obtained on PE/CA-PJ49 tumor cells treated for 24 h with 10 µM CisPt applied alone showed that *MDM-2* gene expression was not modified compared to untreated cells, while 50 µM RSV induced a significant increase of the *MDM-2* gene expression (*p* < 0.0005, ***), compared to the effect induced by CisPt. Additionally, it was observed that the combined treatment CisPt + RSV amplified the *MDM-2* gene expression in PE/CA-PJ49 tumor cells more than the effect induced by CisPt alone (*p* < 0.003, **) or RSV alone (*p* < 0.004, **) (Figure 2B, Table 3).

Treatment with 50 µM RSV (*p* < 0.0025, **) applied alone on the HaCaT normal cell line induced a significant increase of *MDM-2* gene expression compared to untreated cells (control) and caused a much bigger effect than that induced by treatment with 10 µM CisPt (*p* < 0.013, *) versus control (Figure 2B, Table 3). The effect of CisPt + RSV treatment seems to increase *MDM-2* gene expression, being significantly stronger compared to the effect induced by treatment with CisPt alone (*p* < 0.001, **) (Figure 2B, Table 3).

#### 2.2.3. *BCL-2* Gene Expression Analysis

CisPt did not affect *BCL-2* gene expression in HaCaT normal cells. Treatment with CisPt significantly reduced *BCL-2* gene expression in FaDu (*p* < 0.05, *) and PE/CA-PJ49 (*p* < 0.025, *) tumor cell lines compared to the effect induced on the HaCaT normal line (Figure 2C, Table 3).

As shown in Figure 2C, treatment with RSV acted similarly on FaDu tumor cells (*p* < 0.005, **) and HaCaT cells (*p* < 0.006, **), and it resulted in the inhibition of *BCL-2* gene expression compared to untreated cells. RSV did not significantly modify *BCL-2* gene expression in PE/CA-PJ49 tumor cells, the effect being different from the one induced in HaCaT cells (*p* < 0.035, *). Furthermore, by comparing the level of expression of the *BCL-2* gene in the two tumor lines, PE/CA-PJ49 and FaDu (*p* < 0.03, *), the different action mode of RSV was observed (Figure 2C, Table 3). The expression of the *BCL-2* gene in FaDu tumor cells was less affected by combined treatment with CisPt + RSV (*p* < 0.04, *), compared to the effect induced by RSV alone. In PE/CA-PJ49 tumor cells, combined treatment with CisPt + RSV (*p* < 0.05, *) decreased *BCL-2* gene expression compared to the effect induced by RSV applied alone.

Thus, it was seen that the CisPt effect appeared to be dominant in the decreasing of *BCL-2* expression when the combined treatment CisPt + RSV was applied. Analysis of *BCL-2* gene expression in HaCaT normal cell line showed that the effect induced by the combined treatment CisPt + RSV was significantly different than the one with only CisPt (*p* < 0.03; *) (Figure 2C, Table 3). Comparative analysis of *BCL-2* gene expression in PE/CA-PJ49 and FaDu cell lines showed that these tumor cells had a similar response to treatment with CisPt and to the combined treatment CisPt + RSV, but they reacted in a different way to treatment with RSV alone (*p* < 0.03, *).

#### 2.2.4. *c-MYC* Gene Expression Analysis

*c-MYC* plays an important role in the progression of the cell cycle, apoptosis, and malignant transformation. The association of the *c-MYC* gene with tumor progression can partially be explained by its role in regulating the cell cycle, as it is a regulator of the cyclin-dependent kinases. An increased level of *c-MYC* expression has been observed in many different types of cancer, including HNSCC cancers [71,72]. It is known that *c-MYC* can participate in the process of tumor initiation, but it has not yet been discovered if it is involved in tumor progression or the response to therapy. The hypothesis of our study was to modulate the *c-MYC* gene expression through treatment with CisPt associated with a natural compound such as RSV.

Treatment with 10 µM CisPt on a HaCaT normal cell line did not affect the *c-MYC* gene expression, but treatment with 50 µM RSV (*p* < 0.01, *) reduced the gene’s expression compared to the control. Also, the treatment of HaCaT cells with CisPt + RSV did not lead to significant changes in *c-MYC* gene expression (Figure 2D, Table 3). Treatment with 10 µM CisPt resulted in a reduction of *c-MYC* gene expression in FaDu (*p* < 0.01, *) and less in PE/CA-PJ49 tumor cells compared to the control (untreated cells) (Figure 2D, Table 3). RSV did not significantly affect the expression of *c-MYC* in PE/CA-PJ49 tumor cells, but it resulted in a significant reduction of *c-MYC* expression in FaDu tumor cells compared to the control (*p* < 0.002, **) or to PE/CA-PJ49 tumor cells (*p* < 0.025, *) (Figure 2D, Table 3).

Combined treatment with CisPt + RSV acted differently on *c-MYC* expression in the two tumor cell lines. Therefore, in the case of the PE/CA-PJ49 tumor line, combined treatment with CisPt + RSV (*p* < 0.02, *) amplified the effect induced by CisPt, and it resulted in the reduction of *c-MYC* expression compared to the effect induced by RSV.

The response of FaDu tumor cells to combined treatment with CisPt + RSV led to the significant amplification of *c-MYC* gene expression, an antagonist effect to the one induced by CisPt (*p* < 0.009, **) or RSV (*p* < 0.007, **) used separately (Figure 2D; Table 3).

### 2.3. The Role of RSV in Modulating the Apoptotic Process of Head and Neck Tumor Cells

In this study, we analyzed the effects induced by RSV on PE/CA-PJ49 and FaDu tumor cells treated with CisPt, in order to reduce the side effects generated by treatment with a cytostatic agent and to increase the efficiency of the cellular response to conventional therapy. Analysis of apoptotic events in PE/CA-PJ49 and FaDu tumor lines, as well as in the HaCaT control cell line, was performed using the Annexin V-FITC Apoptosis Detection Kit I (BD Bioscience Pharmingen, USA), which ensures the double labeling of the cells with annexin V-FITC and propidium iodide (PI). Data were acquired with a BD FACS Canto II flow cytometer using specific acquisition and data analysis programs. Thus, cells positive for annexin V and negative for PI were considered early apoptotic events (Q4 lower right quadrant), while double-positive cells for annexin V and PI were considered late apoptotic events (Q2), and cells positive only for PI were considered necrotic (Q1 upper right quadrant). Cells in Q3 represent living cells.

**Figure 3:**
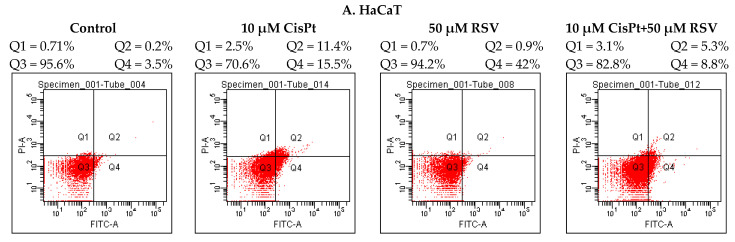

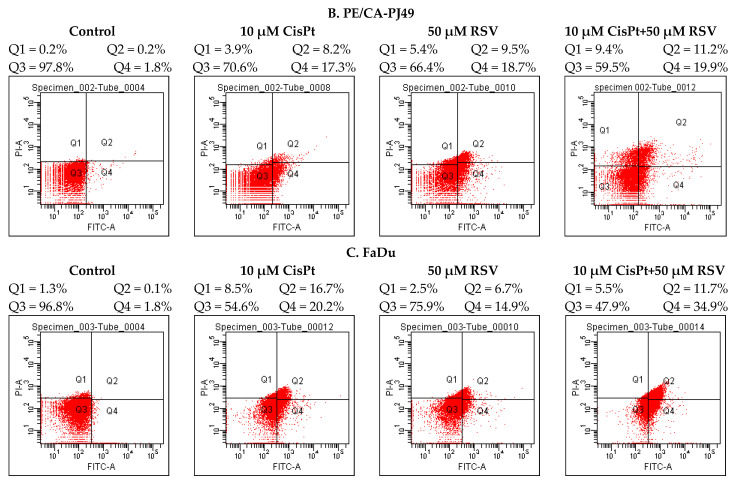
Apoptosis of PE/CA-PJ49 (**B**) and FaDu (**C**) tumor cells versus HaCaT (**A**) normal cells induced by 24 h treatment with RSV and/or CisPt.

**Figure 4:**
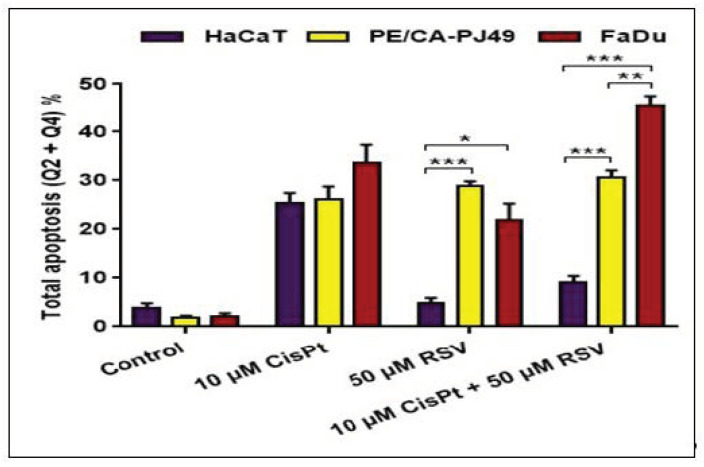
The effect of RSV on the apoptotic process of PE/CA-PJ49 and FaDu tumor cells versus HaCaT normal cells treated 24 h with CisPt. (* *p* < 0.05, ** *p* < 0.005, *** *p* < 0.0005).

In order to analyze the effects induced by treatment with CisPt and/or RSV on the apoptotic process in normal human cells, we used the HaCaT cellular line, which we subjected to a treatment regimen similar to the one used in the case of PE/CA-PJ49 and FaDu tumor cell lines (Figure 3 and Figure 4). The results showed that treatment with RSV did not affect HaCaT control cells, while CisPt induced a significant increase of the apoptotic process (*p* < 0.0001, ***) compared to untreated cells. Combined treatment (CisPt + RSV) (*p* < 0.003, **) induced apoptosis of HaCaT cells in a much lower percentage than that induced by CisPt. These data suggest the capacity of RSV to protect normal cells from the effect induced by CisPt (Figure 3 and Figure 4).

In PE/CA-PJ49 tumor cells treatment with RSV (*p* < 0.00002, ****) induced apoptosis in a similar manner to CisPt (*p* < 0.00001, ****) compared to untreated cells (control). Simultaneous treatment with CisPt + RSV on PE/CA-PJ49 tumor cells did not modify the apoptotic process compared to the response to treatment with CisPt (*p* < 0.1, ns) or RSV (*p* < 0.4, ns) alone.

In FaDu tumor cells, CisPt (*p* < 0.00001, ****) or RSV (*p* < 0.0001, ***) applied separately induced a significant increase of the apoptotic process compared to untreated cells (control). Comparative analysis of the effect induced by RSV versus CisPt applied individually showed that RSV activated the apoptotic process of FaDu tumor cells in a slightly different way than CisPt (*p* < 0.016, *). When the two agents CisPt + RSV were applied simultaneously, a significant increase of the apoptotic process took place compared to the effect induced by CisPt alone (*p* < 0.0007, ***) and by RSV alone (*p* < 0.0002, ***). Enhanced apoptosis in FaDu cells treated with CisPt + RSV compared to untreated cells (*p* < 0.000002, *****) supported the modulatory effect of RSV on apoptosis by potentiating the effect induced by treatment with CisPt alone.

### 2.4. The Role of RSV in Modulating the Cell Cycle of Head and Neck Tumor Cells

**Figure 5:**
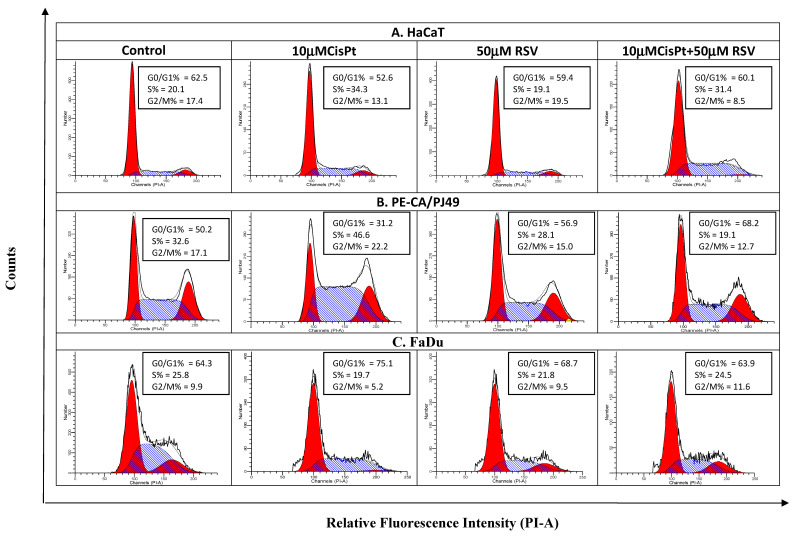
The effect of the treatment with CisPt and/or RSV on cell cycle phase distribution in normal cells HaCaT (**A**), PE/CA-PJ49 (**B**), and FaDu tumor cells (**C**) flow cytometry histograms.

**Figure 6:**
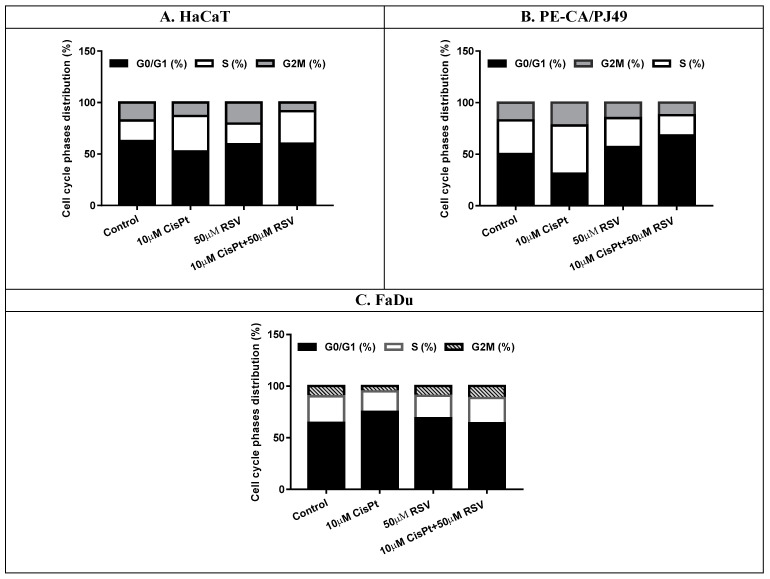
Cell cycle phase distribution (%) in normal HaCaT cells (**A**) and tumor cells: PE/CA-PJ49 (**B**) and FaDu (**C**), after treatment with CisPt and/or RSV.

Rigorous control of cellular proliferation and the differentiation process is necessary to ensure the normal growth and development of the human body. Any alteration of the cellular division pathways leads to the formation of tumors and the appearance of the carcinogenetic process. Cells from the HaCaT cell line used as control presented a 20% distribution of the cells in the synthesis phase (S). Treatment with 10 μM CisPt determined an increase of the synthesis phase to 34.3% in HaCaT cells compared to 20.1% registered in untreated cells (Control). These data showed that treatment with CisPt affected the development of the cell cycle in HaCaT normal cells. Treatment of HaCaT cells with 50 μM RSV did not seem to significantly affect the distribution of the cellular cycle when applied alone and it did not influence the effect induced by treatment with CisPt when treatment with RSV + CisPt was applied simultaneously (Figure 5 and Figure 6).

Analysis of data obtained in the case of PE/CA-PJ49 tumor cells highlighted a different effect than the one found in FaDu tumor cells. Therefore, treatment with 10 μM CisPt in PE/CA-PJ49 tumor cells resulted in a decrease of the G0/G1phase (31.2% versus control 50.2%), accompanied by a slight increase of the synthesis phase (46.6% versus control 32.6%), alongside a lower increase of the G2 + M phase (22.2% versus control 17.1%) (Figure 5 and Figure 6). The effects induced by treatment with 50 μM RSV on PE/CA-PJ49 showed a slight decrease in the % of the cells in the synthesis phase (28.1% versus control 32.6%) and an increase of the % of cells in the G0/G1 phase (56.9% versus control 50.2%). The combined treatment CisPt + RSV applied on PE/CA-PJ49 resulted in an increase in the % of cells in the G0/G1 phase (68.2% versus control 50.2%) and a decrease in the proportion of cells in the synthesis phase (19.1% versus control 32.6%). The results presented in Figure 5 and Figure 6 show that, although RSV alone acted on the cell cycle in a different manner than CisPt on the PE/CA-PJ49 cells, when RSV + CisPt were used together the dominant effect seemed to be that of RSV, registering a decrease of the % of cells in the synthesis phase associated with the blocking of the cells in the G0/G1 phase.

Data analysis highlighted the fact that treatment with 10 μM CisPt in FaDu tumor cells caused a decrease of the % of cells in the synthesis phase (19.7% versus control 25.8%) simultaneously with the growth of the proportion of cells in the G0/G1 phase (75.1% versus control 64.3%) (Figure 5). Treatment with 50 μM RSV seemed to slightly reduce the cells in the synthesis phase in FaDu cells (21.8% versus control 25.8%), accompanied by a slight growth of the % of cells in the G0/G1 phase (68.7% versus control 64.3%). Data showed that RSV acted similarly to CisPt and slightly reduced the % of cells in the synthesis phase (21.8% versus control 25.8%). When RSV was applied together with CisPt in FaDu cells we did not record changes in the cell cycle.

## 3. Discussion

The side effects induced by chemotherapeutic agents along with the risk of drug resistance are the obstacles often encountered in the clinic for patients with head and neck malignancies. To reduce the side effects generated by CisPt, we used RSV, a natural product known for its antioxidant and anti-tumor properties.

The data obtained by analyzing the proliferative process induced by RSV and/or CisPt showed that 50 µM RSV inhibited the proliferation of FaDu tumor cells much more than that of PE/CA-PJ49 tumor cells, but the proliferative process was less affected in normal HaCaT cells. The combined treatment with 10 µM CisPt + 50 µM RSV influenced the proliferative process in a slightly different manner in the two tumor cell lines. Treatment with 10 µM CisPt + 50 µM RSV reduced the proliferation of PE/CA-PJ49 tumor cells, but the dominant effect on proliferation was determined by CisPt, while in FaDu tumor cells RSV amplified the proliferation inhibition induced by CisPt. In addition, when cells were treated simultaneously with 10 μM CisPt + 50 μM RSV, the RSV had a protective effect against the toxic effect induced by CisPt in normal HaCaT cells.

Unlike normal cells, which, as soon as DNA damage is detected, act immediately to repair it or activate the apoptotic process, in tumor cells these mechanisms of repair or induction of apoptosis are defective and allow excessive cell proliferation. Therefore, we analyzed the effect of RSV on the gene expression process for some genes involved in the apoptotic process or involved in cell cycle blocking in FaDu and PE/CA-PJ49 tumor cells versus normal HaCaT cells treated or not treated with CisPt.

The first gene expression level analyzed was the *TP53*, which in normal HaCaT cells treated with RSV or CisPt was slightly low, but when the combined treatment CisPt + RSV was applied an increase (3X) in the *TP53* gene expression was recorded, as compared to untreated cells or cells treated separately with CisPt or RSV (Figure 2A). The results showed that in FaDu cells treatment with CisPt and/or RSV acted in the sense of amplifying the expression of the *TP53* gene, while in PE/CA-PJ49 tumor cells the combined treatment CisPt + RSV led to a decrease of the *TP53* gene expression in an antagonist way as compared to the response to CisPt or RSV applied individually (Figure 2A).

The *MDM-2* gene is considered to be a factor involved in the inhibition of the p53 pathway in tumor cells and, therefore, we analyzed how its gene expression is affected by RSV and/or CisPt treatment. *MDM-2* gene expression in FaDu cells treated with CisPt and/or RSV appeared to be slightly inhibited. RSV induced in PE/CA-PJ49 cells about 8X amplification of *MDM-2* gene expression associated with a slight increase in *TP53* expression (1.5X) (Figure 2B). The combined CisPt + RSV treatment appeared to cause an increase (4X) in *MDM-2* gene expression (Figure 2B) but lower as compared to the RSV-induced effect, and this could be associated with decreased *TP53* gene expression (Figure 2A) recorded in CisPt + RSV-treated PE/CA-PJ49 cells.

Comparative analysis of CisPt or the effects induced by RSV applied separately versus CisPt + RSV combination therapy showed that RSV was responsible for modulating *MDM-2* gene expression in PE/CA-PJ49 tumor cells. The data obtained showed that *MDM-2* gene expression in both tumor cell lines was influenced in a different way (Figure 2).

*BCL-2* is another gene responsible for inhibiting apoptosis and promoting aberrant cell proliferation, so we analyzed how its expression was modulated in tumor cells treated with RSV + CisPt. Comparative analysis of *BCL-2* gene expression in the PE/CA-PJ49 and FaDu cell lines showed that the two types of tumor cells had a similar response to CisPt treatment as well as to the combined CisPt + RSV treatment. RSV treatment applied alone influenced the response of tumor cells in a different way, thus a significant decrease in *BCL-2* gene expression in FaDu was recorded without altering the expression in PE/CA-PJ49 cells. The reduced *BCL-2* gene expression in FaDu tumor cells could explain the high percentage of apoptosis recorded by RSV alone or RSV + CisPt-treated cells.

Different molecular mechanisms mediate the processes of hyperproliferation and apoptosis, but it seems that the *c-MYC* gene has a decisive role in the cell choice between blocking the cell cycle or entering apoptosis. For this reason, we analyzed how treatment with RSV and/or CisPt affected *c-MYC* expression in tumor cells as compared to normal cells. The modulation of *c-MYC* gene expression, when treated with CisPt and/or RSV, was different in the two tumor cell lines. In the FaDu line, both CisPt and RSV administered separately caused a reduction of *c-MYC* gene expression, while in the case of combined treatment an amplification of gene expression was registered (2X) (Figure 2D; Table 3). In PE/CA-PJ49 tumor cells treatment with RSV alone acted in an opposite way to the effect induced by CisPt alone on *c-MYC* gene expression. When the combination of the two agents was used, RSV amplified the effect induced by CisPt and induced a significant inhibition of *c-MYC* expression. In normal HaCaT cells, RSV + CisPt did not significantly affect *c-MYC* gene expression. Data obtained showed that RSV can modulate the expression of the analyzed genes in a different way, depending on the morphological, functional, and molecular features of the tumor cell line studied.

Apoptosis is a program that removes damaged cells and thus ensures the regression of tumors after chemotherapy. Tumor suppression is provided by complex networks that include many genes involved in the induction of apoptosis [73]. Therefore, an attempt was made to correlate the level of expression of some genes with the apoptotic process induced by treatment with CisPt and RSV to decipher how tumor cells respond to therapy. Investigations regarding the effects of resveratrol on apoptosis induction in tumor cells FaDu and PE/CA-PJ49 revealed that RSV can stimulate apoptosis by modulating the expression of some genes (*TP53*, *MDM-2*, *BCL-2*, *c-MYC*).

The percentage of normal HaCaT cells entered in apoptosis as a result of treatment with CisPt alone or combined with RSV showed that CisPt affected HaCaT normal cells more than RSV. The high percentage of HaCaT cells that entered apoptosis after treatment with CisPt + RSV was associated with increased expression of *TP53* and *MDM-2* genes and a slight increase in the S phase of the cell cycle. In conclusion, when CisPt + RSV were used together, RSV seemed to protect HaCaT normal cells from the effect induced by CisPt (Figure 3 and Figure 4).

Treatment with CisPt or RSV alone on PE/CA-PJ49 tumor cells caused the cells to enter apoptosis, but in the case of the combined treatment CisPt + RSV, the two agents did not have an additive effect on the apoptotic process. In the case of CisPt + RSV combination therapy, an increase in the percentage of cells in apoptosis appeared to be associated with inhibition of *BCL-2* gene expression. The apoptotic pathway involving the activation of the *TP53* gene appeared to be inhibited by the exaggerated increase in *MDM-2* gene expression (Figure 2B). The important role in inducing apoptosis in PE/CA-PJ49 tumor cells can be attributed to RSV. Data related to the effect of CisPt and/or RSV treatment on the cell cycle showed that cells were blocked in the G0/G1 phase, accompanied by a decrease in phase synthesis associated with decreased *c-MYC* gene expression. These correlations showed that when *c-MYC* gene expression was diminished by treatment with CisPt and/or RSV (Figure 2D), the apoptotic process was hampered, and we can consider this a weaker response of PE/CA-PJ49 tumor cells to CisPt (Figure 4).

In FaDu tumor cells the apoptotic process induced by treatment with CisPt was more pronounced than in the case of treatment with RSV. When the cells were being treated with CisPt + RSV almost 50% of FaDu cells entered in apoptosis, suggesting RSV’s role in potentiating the effect induced by treatment with CisPt in FaDu cells. The increase in FaDu cell apoptosis was directly proportional to the increase in expression of the *c-MYC* and *TP53* genes induced by the combined CisPt + RSV treatment. RSV acted similarly to CisPt, and it slightly reduced the synthesis phase of the FaDu cells but did not affect the cell cycle phases when applied together with CisPt. Thus, the increase of the *c-MYC* expression can be associated both with the activation of the apoptotic process and with the increase of the sensitivity to CisPt as a result of its association with RSV.

In conclusion, although the analyzed cellular and molecular processes showed different modes of action of RSV depending on the particularities of the analyzed tumor cells, the results sustained the role of RSV to potentiate the response to CisPt therapy by sensitizing tumor cells to enter into apoptosis or to block the cell cycle. Furthermore, the results showed that RSV can be considered a useful adjuvant in cisplatin therapy because it increases the sensitivity of head and neck tumor cells to cisplatin. Also, the way in which RSV influences tumor cells to enter the apoptotic process or to repair the DNA damages and to continue the cellular cycle depends on the level of gene expression, as well as on the morpho-functional characteristics of the studied tumoral cells.

Data provided by experiments performed on the normal HaCaT line demonstrated the role of RSV in chemoprevention by modulating cell proliferation or cell cycle and by reducing the effects of CisPt in normal cells.

Finally, on the studied head and neck tumor cells, resveratrol, in addition to its anti-proliferative activity, facilitated the induction of apoptosis by CisPt, which could reduce the risk of cell resistance to CisPt treatment. These promising results will encourage the expansion of the studies on animal models and primary cells obtained from patients with head and neck tumors.

## 4. Materials and Methods

### 4.1. Materials

Resveratrol (RSV) and Cisplatin (CisPt), dimethyl sulfoxide (DMSO), trypsin-EDTA (0.25% trypsin, 0.03% EDTA), Glutamine (Glu), Dulbecco’s modified Eagle’s medium (DMEM), fetal bovine serum (FBS), and 3-(4,5-dimethylthiazol-2-yl)-2,5- diphenyltetrazolium bromide (MTT), phosphate-buffered saline (PBS) pH7.2–7.4; Propidium Iodide (PI) stock solution: 4 mg/mL PI in PBS; RNase A stock solution: 10 mg/mL RNase A were purchased from Merck KGaA, Saint Louis, MO, USA.

Annexin V-FITC kit (BD Biosciences, San Jose, CA 95131, USA); High-capacity cDNA Reverse Transcription Kits (Applied Biosystems, Beverly Hills, CA, USA) with the following components: RT-Buffer 10×, 1 mL; RT- 10× random primers, 1 mL; 25× dNTP mix (100 mM); MultiScribe™ Reverse Transcriptase, 50 U/uL; RNaza inhibitor 100 uL; Ultrapure water; validated TagMan probes: *TP53* (Hs01034249_m1); BCL-2 (Hs00153350_m1); *MDM-2* (Hs01066930_m1); MYC (Hs00153408_m1); HPRT1 (Hs02800695_m1); qPCR master mix; plates (MicroAmp Fast Optical 96-Well Reaction Plate, 0.1 mL).

#### Stock Solutions

Resveratrol and cisplatin were dissolved in dimethyl sulfoxide (DMSO) at a concentration of 50 mM and then a 1 mM stock solution was prepared using Milli-Q water. The stock solutions were filtered using a cellulose acetate hydrophilic filter (0.20 μm) (Merck KGaA (Saint Louis, MO, USA), and used to obtain the working concentrations of 10 μM for CisPt and 50 μM for RSV by performing dilutions in the culture medium.

### 4.2. Experimental Methods

#### 4.2.1. Cell Lines Culture

The squamous tongue carcinoma cell line PE/CA-PJ49 was from the European Collection of Authenticated Cell Cultures (ECACC cat. no. 0060606, Culture Collections Public Health England, Porton Down, Salisbury, SP4 0JG, UK), the squamous pharyngeal carcinoma cell line FaDu was obtained from the American Type Culture Collection (Cat. no ATCC-HTB-43, ATCC Manassas, VA, USA), HaCaT cells, immortalized human keratinocyte line, were provided by Cell Line Service GmbH (Cat no.330493, Eppelheim, Germany) and were grown and maintained in Dulbecco’s modified Eagle’s medium (DMEM) supplemented with 10% fetal bovine serum (FBS), 2 mM glutamine and then maintained at 37 °C in a 5% CO_2_ humidified incubator. The cell lines showing adherent epithelial type morphology and the subconfluent cultures (70–80%) were split 1:4-1:8 (i.e., seeding at 1–3 *×* 10,000 cells/cm^2^) using trypsin-EDTA (0.25% trypsin, 0.03% EDTA). The adherent cells were incubated for 24, 48, or 72 h either in the presence of the drugs (CisPt and/or RSV) or in vehicle control (DMSO ≤ 0.1%). The treated and untreated cells were used to analyze the proliferative process, gene expression levels, apoptosis, distribution of cell cycle phases, or for PCR assays. In all experiments described in this study, all untreated cells were designated as control cells [74].

#### 4.2.2. Cellular Proliferation Assay

Previous to the proliferation test, in order to select the optimal working concentrations, the tumor and normal cells were treated for 24, 48, and 72 h with RSV or CisPt in concentrations ranging from 2 to 256 µM. Cell viability was measured with the 3-(4,5-dimethylthiazol-2-yl)-2,5-diphenyltetrazolium bromide (MTT) colorimetric assay (Merck KGaA, Saint Louis, MO, USA). Subsequently, the cells were incubated for 4 h with 20 µL 5 mg/mL MTT solution at 37 °C. The MTT liquid was aspirated and 100 µL DMSO was added to each well and the plate was shaken for 10 min. The absorbance of each well was determined at a wavelength of 570 nm using a Dynex plate reader (Dynex Technologies, Chantilly, VA, USA). The drug response curve was generated, and IC50 (the concentration required to kill 50% of the cell population) was calculated using GraphPad Prism version 7.00. For the proliferation assay, PE/CA-PJ49, FaDu, and HaCaT cells (5 × 10^3^ cells/well) were seeded in a 96-well culture plate and then treated with 50 µM RSV and/or 10 µM CisPt and incubated at 37 °C with 5% CO_2_ for 24, 48 or 72 h. The cell proliferation performed using the CellTiter 96^®^ AQueous One Solution Cell Proliferation Assay (Promega, Saint Louis, MO, USA) was based on the reduction of yellow MTS tetrazolium salt by the viable cells and generation of colored formazan soluble in the culture medium. The product was spectrophotometrically quantified by measuring the absorbance at λ = 490 nm using a Dynex plate reader (Dynex Technologies, MRS, USA). Results were expressed as mean values of three determinations ± standard deviation (SD). Untreated cells served as control and were considered to have a proliferation index (IP) equal to 1. Index proliferation (IP) was calculated using the optical density (OD) as follows: IP = (ODtreatment−ODblank)/(ODcontrol−ODblank) [75].

#### 4.2.3. Real-Time PCR for Analysis of Gene Expression

A number of 5 × 10^6^ cells treated with 50 µM RSV and/or 10 µM CisPt for 24 h were used to extract total RNA with Trizol Reagent (Invitrogen, Carlsbad, CA, USA) as indicated in the protocol. The concentration of isolated RNA was assessed using a NanoDrop spectrophotometer (NanoDropTechnologies, Montchanin, DE, USA). RNA purity was determined by A260/A280 ratios (an A260/A230 ratio as close as possible to 2 indicates the presence of highly purified RNA). For each sample, 2 µg of total RNA was reverse-transcripted using the High-capacity cDNA Reverse Transcription Kit from Applied Biosystems, Beverly Hills, CA, USA, using non-specific, random primers, following the manufacturer’s instructions. The obtained cDNA was stored at 4 °C and 50 ng of cDNA from each sample was used in the amplification reaction real-time PCR (RT-PCR).

The analysis of the gene expression level (TP53, MDM-2, BCL-2, MYC) was performed by real-time PCR using a ViiA™ 7 Real-Time PCR System by setting the ABI 7500 Fast program (Applied Biosystems, Beverly Hills, CA, USA). The reference gene used in the experiments was HPRT1 (hypoxanthine ribosyltransferase 1) because this gene is found in all cell types and has a stable, relatively constant expression regardless of experimental conditions. The reference gene is useful in calibrating and interpreting RT-PCR [76,77]. Each sample was performed in duplicate. Thermal cycling conditions of PCR were as follows: 95 °C for 10 min for amplification activation and 40 cycles at 95 °C for 12 s and 60 °C for 15 s. The amplification was examined with the use of the 7300 Real-Time PCR System-SDS, Version 1.4, program. Results were analyzed with Relative Quantitation RQ study software (Applied Biosystems) applying the Delta-Delta-Ct (ΔΔCt) Algorithm an approximation method to determine relative gene expression with RT-PCR experiments, in order to collect, analyse and visualize RT-PCR results. The 2-ΔΔCt value obtained indicates how many times the expression of the gene has increased or decreased compared to the control sample (untreated cells).

#### 4.2.4. Flow Cytometry Methods

- Apoptosis Analysis
  The apoptosis assay was carried out with the Annexin V-FITC Apoptosis Detection Kit (BD Biosciences, San Jose, CA 95131, USA) according to manufacturer protocol. The cells were cultured for 24 h and treated with 10 μM CisPt and/or 50 μM RSV for another 24 h. Then, treated and untreated cells were detached using trypsin-EDTA (0.25%) solution, centrifugated 10 min at 200× *g*, and were resuspended in cold binding buffer and staining simultaneously with 5 μL FITC-Annexin V (green fluorescence) and 5 μL propidium iodide (PI) in the dark, at room temperature for 15 min. A total of 400 μL of Annexin V binding buffer was added and 10,000 cells/sample were acquired using a BD Canto II flow cytometer. The analysis was done using DIVA 6.2 software to discriminate viable cells (FITC^−^PI^−^) from necrotic cells (FITC^+^PI^+^) and early apoptosis (FITC^+^PI^−^) from late apoptosis [78].
- Cell Cycle Analysis by Flow Cytometry
  For analysis of cell cycle distribution, the treated and untreated cells were harvested, washed in a cold PBS solution, and fixed with 70% ethanol for at least 24 h at 4 °C. After 24 h, the cells were stained with propidium iodide (PI), an agent that intercalates into the major groove of double-stranded DNA and produces a highly fluorescent adduct that can be excited at 488 nm with a broad emission around 600 nm. Since PI can also bind to double-stranded RNA, it is necessary to treat the cells with RNase for optimal DNA resolution. Cells (1 × 10^6^ cells/mL) were washed in PBS and centrifuged at 300× *g*, 5 min at 4 °C. The pellet of the cells was incubated for 10 min at 37 °C with 0.5 mg/sample RNase A and then 10 μg/sample of PI staining solution was added to cell pellet, mixed well, and incubated 10 min at 37 °C. The samples were stored at 4 °C until analyzed by flow cytometry. A minimum of 20,000 events for each sample was collected using a FACS CantoII flow cytometer and ModFIT software (BD Biosciences, San Jose, CA 95131, USA) and used to determine the cell cycle phase distribution after debris exclusion [79].
- Statistical Analysis
  All data analyses were performed using GraphPad Prism 7 (GraphPad Software Inc., La Jolla, CA, USA). The differences between the treatment and control groups or between different treatments were statistically analyzed using unpaired two-tailed t-test and one-way ANOVA. Statistical significance was considered at *p* < 0.05.

## Figures and Tables

**Table 1:** IC50 (inhibitory concentration) values of the cisplatin and resveratrol were determined using a linear regression equation for the cytotoxicity curve for PE/CA-PJ49, FaDu tumor cells and for HaCaT normal cells. IC50 values are presented as mean ± SEM according to two independent assays, each done in triplicate. The Selectivity Index (SI) = IC50 of a compound in a normal cell line/IC50 of the same compound in a cancer cell line. SI values of more than 2 were considered as high selectivity.

	IC50			SI	
24 h Treatment	HaCaT	PE/CA-PJ49	FaDu	HaCaT/PE/CA-PJ49	HaCaT/FaDu
CisPt (µM)	23.9+/−1.6	9.1+/−2.1	12.1+/−1.9	2.63	1.97
RSV (µM)	94.7+/−2.9	44.9+/−3.1	39.3+/−1.9	2.15	2.41

**Table 2:** The index of proliferation (IP) for tumor cell line PE/CA-PJ49, FaDu, and normal cell line HaCaT treated with RSV and/or CisPt for 24, 48, and 72 h. IP is equal to absorbance of treated cells/absorbance of untreated cells. Results are expressed as mean values of three determinations ± standard deviation (SD). Untreated cells serve as control and are considered to have the index of proliferation (IP) equal to 1.

Treatment/Cell Lines	HaCaT			PE/CA-PJ49			FaDu		
	24 h	48 h	72 h	24 h	48 h	72 h	24 h	48 h	72 h
10 µM CisPt	0.52	0.35	0.56	0.55	0.53	0.50	0.67	0.55	0.51
50 µM RSV	0.79	0.42	0.81	0.68	0.64	0.60	0.53	0.49	0.57
10 µM CisPt + 50 µM RSV	0.74	0.68	0.70	0.43	0.48	0.52	0.59	0.53	0.51

**Table 3:** n-fold gene expression in PE-CA/PJ49 or FaDu tumor cells versus normal HaCaT cells treated for 24 h with 10 μM CisPt and/or 50 μM RSV. The results were obtained using gene reference HPRT. Control is represented by the untreated cells of each cell line, and the n-fold expression for each gene analyzed in control cells was 1.

n-Fold Gene Expression/Treatment	HaCaT			PE/CA-PJ49			FaDu		
	CisPt	RSV	CisPt + RSV	CisPt	RSV	CisPt + RSV	CisPt	RSV	CisPt + RSV
TP53	0.69	0.82	3.05	1.18	1.44	0.59	2.7	2.5	2.4
MDM2	1.7	5.3	7.3	1.2	7.9	4.2	0.65	0.46	0.37
BCL2	0.98	0.27	0.62	0.29	0.96	0.59	0.46	0.33	0.17
c-MYC	0.84	0.47	0.74	0.75	1.11	0.44	0.45	0.22	2.3
